# Mechanical Response of He-Implanted Amorphous SiOC/Crystalline Fe Nanolaminates

**DOI:** 10.1038/s41598-019-41226-w

**Published:** 2019-03-18

**Authors:** A. Zare, Q. Su, J. Gigax, T. A. Harriman, M. Nastasi, L. Shao, D. A. Lucca

**Affiliations:** 1 School of Mechanical and Aerospace Engineering, Oklahoma State University, Stillwater, OK 74078 USA; 2 Nebraska Center for Energy Sciences Research, University of Nebraska-Lincoln, Lincoln, NE 68583 USA; 3 Department of Nuclear Engineering, Texas A&M University, College Station, TX 77840 USA; 4 Department of Mechanical and Materials Engineering, University of Nebraska-Lincoln, Lincoln, NE 68583 USA; 5 Nebraska Center for Materials and Nanoscience, University of Nebraska-Lincoln, Lincoln, NE 68588 USA

## Abstract

This study investigates the microstructural evolution and mechanical response of sputter-deposited amorphous silicon oxycarbide (SiOC)/crystalline Fe nanolaminates, a single layer SiOC film, and a single layer Fe film subjected to ion implantation at room temperature to obtain a maximum He concentration of 5 at. %. X-ray diffraction and transmission electron microscopy indicated no evidence of implantation-induced phase transformation or layer breakdown in the nanolaminates. Implantation resulted in the formation of He bubbles and an increase in the average size of the Fe grains in the individual Fe layers of the nanolaminates and the single layer Fe film, but the bubble density and grain size were found to be smaller in the former. By reducing the thicknesses of individual layers in the nanolaminates, bubble density and grain size were further decreased. No He bubbles were observed in the SiOC layers of the nanolaminates and the single layer SiOC film. Nanoindentation and scanning probe microscopy revealed an increase in the hardness of both single layer SiOC and Fe films after implantation. For the nanolaminates, changes in hardness were found to depend on the thicknesses of the individual layers, where reducing the layer thickness to 14 nm resulted in mitigation of implantation-induced hardening.

## Introduction

The effect of He implantation on structural materials has been a topic of interest for many years. Irradiation and implantation of crystalline metals with He ions lead to the generation of point defects such as vacancies, self-interstitials, and He interstitials. Other defects such as vacancy clusters, voids, and prismatic dislocation loops can also form as a result of the coalescences of generated point defects. At room temperature, He has a high diffusivity and low solubility in metals. He ions diffusing through the matrix tend to precipitate at nearby sinks (vacancies, dislocations, and grain boundaries). Entrapment of He interstitials in vacancies leads to the formation of He-vacancy clusters, which act as nuclei for the formation of larger He bubbles by absorbing more He interstitials and irradiation-induced vacancies. He bubbles can eventually produce recoil interstitial atoms, lead to swelling, surface blistering, and flaking^1–7^. A major concern associated with crystalline metals implanted with He is the degradation of mechanical properties, most notably an increase in yield strength (or hardness) and loss of ductility, caused by the interaction of dislocations with He bubbles and generated defects^1,3–6^. Grain boundaries and interfaces are efficient sinks for point defects generated as a result of ion irradiation. Therefore, materials with high densities of sinks, or traps for point defects, are promising candidates for developing materials that need to withstand irradiation under harsh conditions^8^. Nanolaminate composites that contain relatively large interface areas between the constituent layers are an example of such materials. Studies of crystalline/crystalline nanolaminates composed of Cu/V^4,5,9^, Fe/W^1^, and Cu/Nb^2,4,10,11^ that were subjected to implantation with He ions revealed the effective role of interfaces in reducing the density of He bubbles and suppressing hardening.

In addition to crystalline/crystalline nanolaminates, there has been increasing interest in developing amorphous/crystalline nanolaminates composed of alternating amorphous and crystalline layers as irradiation tolerant materials. Due to the lack of long-range atomic order in amorphous materials, they undergo fluctuations in free volume or local bonding when subjected to irradiation, rather than the generation and coalescence of point defects^12,13^. Additionally, amorphous materials can accommodate implanted He ions into the free volume present in their structure^8^. Studies have demonstrated that at low irradiation fluences, certain families of amorphous alloys exhibit better irradiation tolerance than crystalline metals, with no signs of He bubble formation, surface blistering, or flaking^8,14^. Formation of He bubbles has been reported in amorphous alloys at higher irradiation fluences^14–16^; however, unlike crystalline metals, the presence of He bubbles in the atomic structure of amorphous alloys could lead to improvements in their ductility^15,16^. Studies on amorphous Cu-Zr/crystalline Cu^8^, amorphous Ta/crystalline Cu^17^, and amorphous Fe-Zr/crystalline Fe nanolaminates^18^ demonstrated that amorphous/crystalline interfaces can be effective at reducing bubble density and hardening. However, irradiation-induced devitrification of the amorphous alloy phase has remained a challenge^8^. Another example of an amorphous material with unique irradiation/implantation stability is amorphous silicon oxycarbide (SiOC). Our previous studies on SiOC demonstrated that He bubble formation can be entirely averted due to the rapid diffusion of implanted ions out of the structure^13,19^. It was also demonstrated that amorphous/crystalline nanolaminates consisting of amorphous SiOC/crystalline α-Fe have desirable structural stability over a range of irradiation/implantation conditions (i.e., ion species, energy, and temperature)^20–23^. Potential applications of these nanolaminates include advanced reactor designs and fuel cycle technologies^20,22,24^. Our studies of the mechanical response of SiOC/Fe nanolaminates, with individual layer thicknesses of 72 ± 10 nm, that were subjected to 3.5 MeV Fe ions to damage levels of 10, 20, or 50 displacements per atom (dpa) demonstrated a lower magnitude of irradiation hardening for the nanolaminate compared to films consisting of only SiOC or Fe^23^. Despite the desirable mechanical properties and irradiation tolerance of the SiOC/Fe nanolaminates, their mechanical response after He implantation has not been reported. The present study aims to address this gap by evaluating the mechanical properties of SiOC/Fe nanolaminates and films consisting of only SiOC or Fe before and after He implantation using nanoindentation and *in-situ* scanning probe microscopy (SPM). The observed changes in mechanical properties are explained by implantation-induced microstructural evolutions characterized with X-ray diffraction (XRD) and transmission electron microscopy (TEM). The role of the amorphous/crystalline interfaces is also investigated by studying nanolaminates with different thicknesses of individual layers.

## Results

### Implantation-induced changes in atomic structure

**Figure 1:**
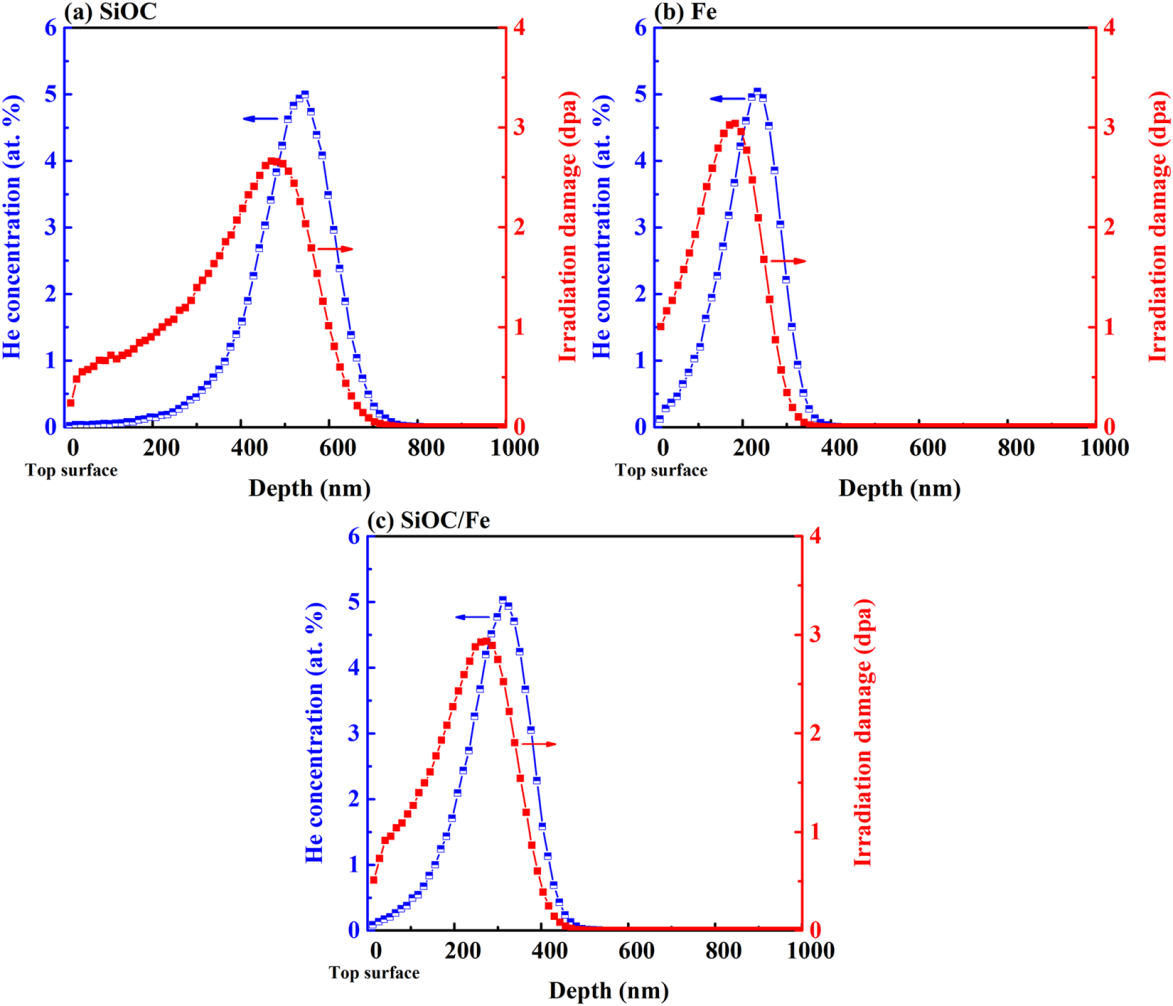
Simulated concentration of implanted He ions and irradiation damage along the thickness of the (**a**) SiOC, (**b**) Fe, and (**c**) SiOC/Fe films.

Figure 1 shows the simulated concentration of implanted He ions and irradiation damage (in dpa) along the thickness of the films. The simulations were performed using the ion distribution and quick calculation of damage option in Stopping and Range of Ions in Matter (SRIM)^25^. The density used for the SiOC was 2.2 g/cm^3^ ^20^ and the displacement energies of Si, O, and C were 15, 28, 28 eV, respectively^23,26^. For Fe, the density used was 6.92 g/cm^3^ ^27^ and the displacement energy was 40 eV^26^. Based on the simulations, the maximum concentration of the implanted He ions was ~5 at. % in all the films, and the maximum irradiation damage was ~2.6 dpa in the SiOC film and ~3 dpa in the Fe film and the SiOC/Fe nanolaminates. Since irradiation damage is primarily produced by nuclear collisions between the incident ions and target atoms^28^, the damage distribution is expected to be closely related to the distribution of implanted ions^29^. However, near the end of the ion range, the ions do not have enough energy to create massive collision cascades^25^. As a result, the simulated depth at which the maximum irradiation damage occurs (474, 185, and 200 nm in the SiOC, Fe, and SiOC/Fe films, respectively), is shallower than the depth corresponding to the maximum concentration of implanted ions (539, 236, and 306 nm in the SiOC, Fe, and SiOC/Fe films, respectively)^29^. Beyond the maxima, both He concentration and irradiation damage decrease and become negligible around a depth of 700, 400, and 450 nm in the SiOC, Fe, and SiOC/Fe films, respectively.

The surface topography and average surface roughness (Ra) of the films before and after implantation was studied by atomic force microscopy (AFM). The obtained AFM images (presented in Supplementary Fig. S1) did not show any evidence of blistering on the surfaces. According to a semi-empirical formula suggested by Wilson^30^, the critical near-surface He concentration (*C*_*He*_) at which blistering occurs in metals is expressed as ${C}_{He}=0.5-(T/{T}_{m})$, where *T* and *T*_*m*_ are the absolute working and melting temperatures. Using this expression at a working temperature of 25 °C, the critical He concentration for the Fe film and the nanolaminates was found to be 34%, which is considerably greater than the concentration of implanted He in the present study (≤5%). The obtained Ra values for the as-deposited SiOC film and the thick and thin SiOC/Fe nanolaminates were similar (≤3 nm) and lower than that of the as-deposited Fe film (~11 nm). Irradiation did not result in a significant change in the Ra values.

**Figure 2:**
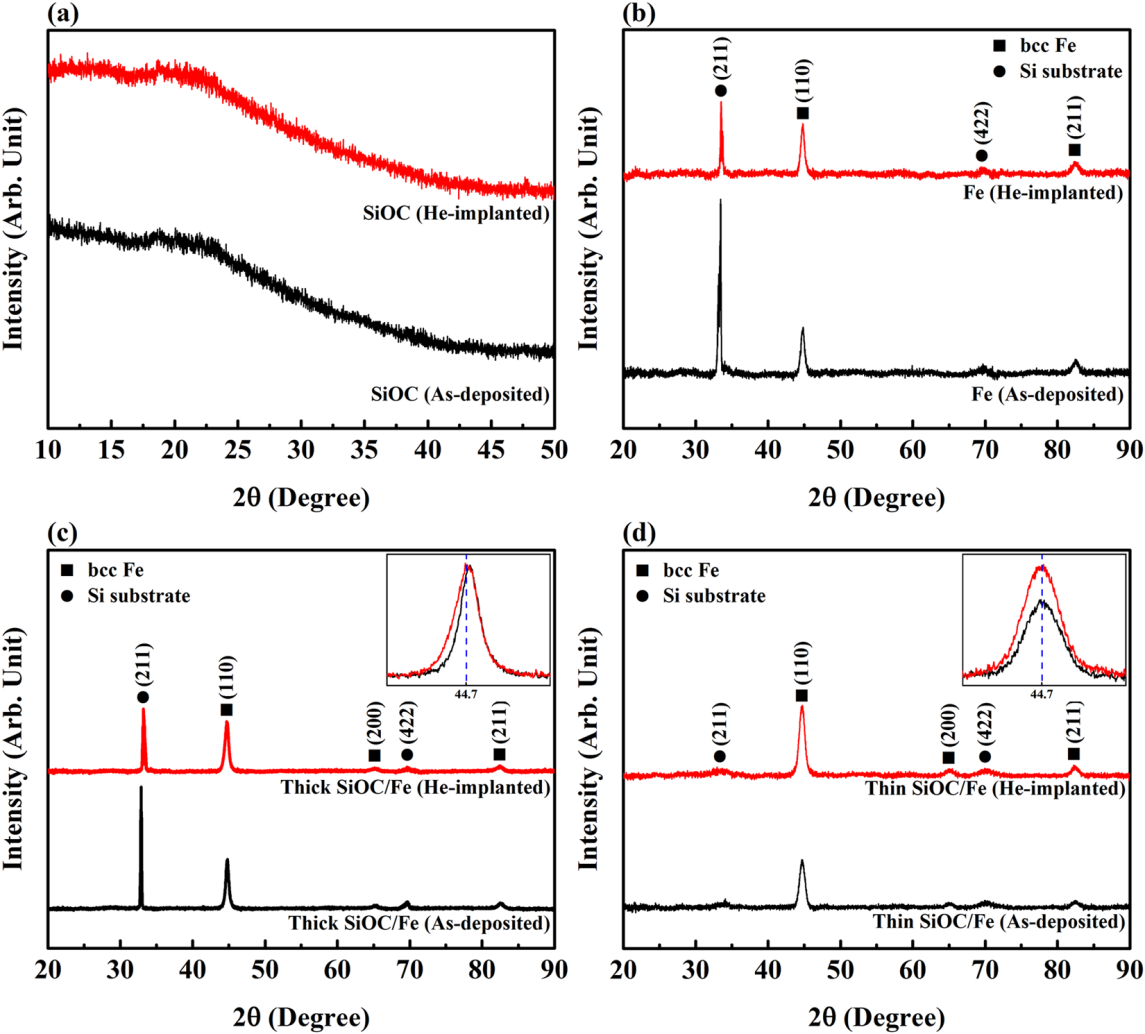
XRD patterns of the (**a**) SiOC, (**b**) Fe, (**c**) thick SiOC/Fe, and (**d**) thin SiOC/Fe films before and after implantation.

The structure of the films before and after implantation was examined by XRD. For the as-deposited and implanted SiOC films, the obtained XRD patterns (Fig. 2a) do not show any crystalline peaks, demonstrating the stability of the amorphous phase after implantation. The XRD patterns of the as-deposited and irradiated Fe films (Fig. 2b) demonstrate the presence of (110) and (211) body centered cubic (bcc) Fe peaks associated with the Fe film, as well as (211) and (422) Si peaks from the substrate. There is no evidence that implantation led to the formation of secondary phases. For the thick and thin SiOC/Fe nanolaminates (Figs. 2c and 2d), the (110), (200), and (211) bcc Fe peaks and (211) and (422) Si substrate peaks are observed in the XRD patterns. There is no evidence that implantation led to phase transformation in the nanolaminates. The XRD patterns of the thick SiOC/Fe nanolaminates indicate a shift of all the bcc Fe diffraction peaks to smaller 2θ values after implantation. An example of this shift for the (110) bcc Fe peak is shown in the inset of Fig. 2c. A shift to a smaller 2θ value has been attributed to an expansion of the lattice spacing^2,5^. XRD patterns of the thin SiOC/Fe nanolaminates do not exhibit any shifts in the diffraction peaks of bcc Fe (see the inset of Fig. 2d).

**Figure 3:**
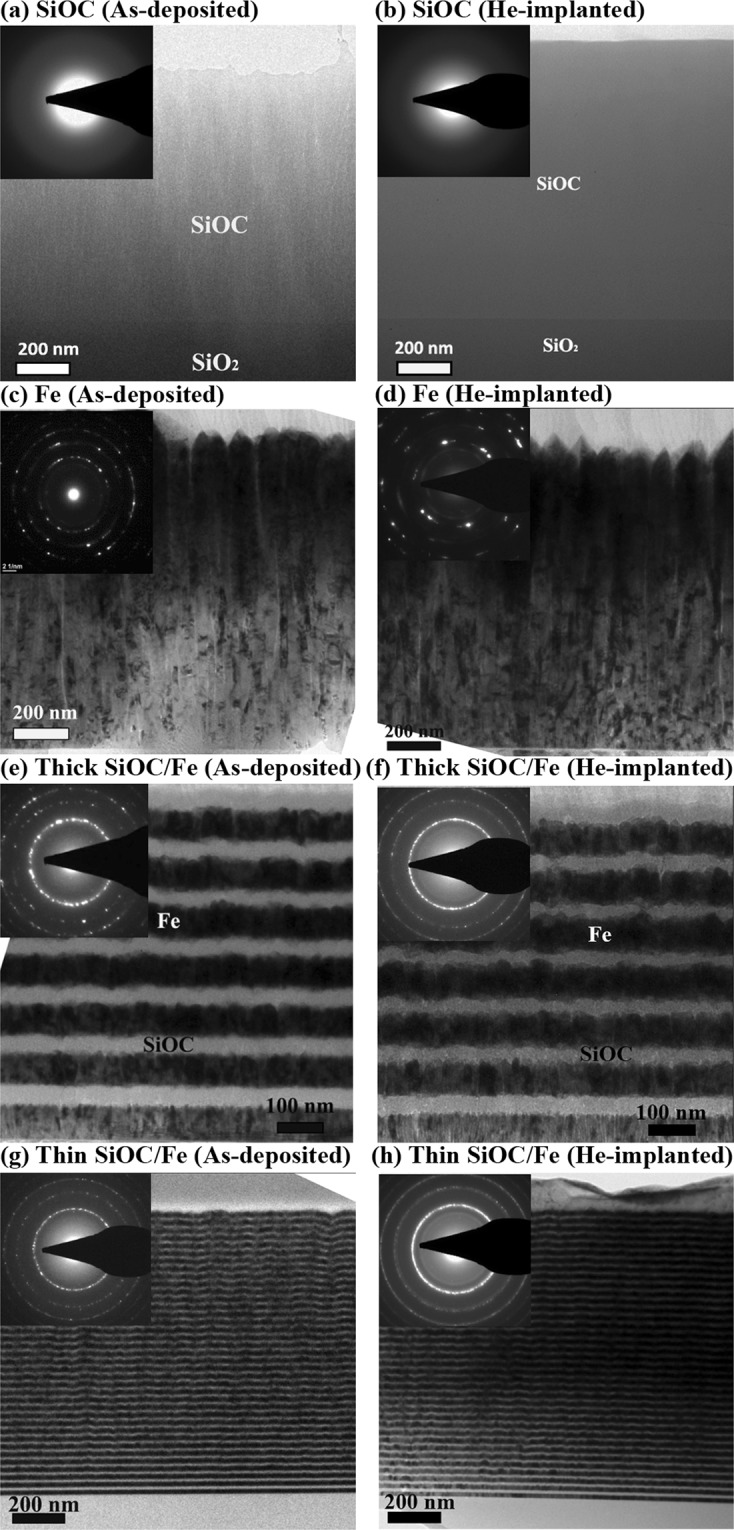
Cross-sectional bright-field TEM micrographs of the as-deposited and implanted films.

Cross-sectional TEM was used to examine the microstructure of the films before and after implantation and to obtain the corresponding selected area diffraction (SAD) patterns. For the as-deposited and implanted SiOC films, the obtained TEM micrographs (Figs. 3a and 3b) exhibit uniform contrast throughout the whole thickness. Consistent with the XRD results, no void formation, segregation, or crystallization is observed after implantation. The corresponding SAD patterns show diffuse halo rings that further confirm that the SiOC retained its amorphous structure after implantation.

An examination of the TEM micrographs of the as-deposited and implanted Fe films (Figs. 3c and 3d) demonstrate that the Fe grains have a columnar structure oriented along the growth direction. Consistent with the XRD results, the SAD patterns reveal the presence of (110) and (211) bcc Fe and no indication of phase transformation after implantation. The average size of the Fe grains was estimated to be 18 ± 5 nm in the as-deposited film. After implantation the average grain size increased to 69 ± 13 nm.

The TEM micrographs of the thick and thin SiOC/Fe nanolaminates before and after implantation are shown in Figs. 3e–3h. The alternating layers of SiOC and Fe have sharp interfaces and uniform thicknesses, although topological irregularities are observed. This is due to the polycrystalline nature of the Fe layers, which caused the subsequently deposited SiOC layers to follow the morphology of the Fe grains^31^. The interfaces between the SiOC and Fe layers remain intact after implantation, indicating a desirable irradiation stability for both nanolaminates. The individual SiOC layers appear to have a uniform contrast with no signs of a secondary phase or voids after implantation. Columnar grains are observed in the Fe layers and their average size (reported in Table 1) is seen to increase after implantation. The corresponding SAD patterns include both a diffuse ring and ring diffraction patterns corresponding to the amorphous SiOC and crystalline Fe layers, respectively. These findings are consistent with the XRD results presented in Figs. 2c and 2d, and also with our previous studies on SiOC/Fe nanolaminates with individual layer thicknesses of 72 ± 10 nm^20,21,23^.

**Table 1:** Grain size, bubble diameter, bubble density, bubble spacing, and changes in hardness for the Fe film and the nanolaminates.

	Fe	Thick SiOC/Fe	Thin SiOC/Fe
Average Fe grain size before implantation (nm)	18 ± 5	16 ± 2	15 ± 2
Average Fe grain size after implantation (nm)	69 ± 13	24 ± 6	17 ± 3
Average bubble diameter (nm)	1 ± 0.2	1 ± 0.1	1 ± 0.1
Maximum bubble density (m^−3^)	2.5 × 10^24^	1.5 × 10^24^	0.9 × 10^24^
Average bubble spacing (nm)	18	25	32
Implantation-induced hardening from FKH model (GPa)	0.3	0.2	0.1
Hardness changes from nanoindentation results (GPa)	0.8	1.4	−0.1

**Figure 4:**
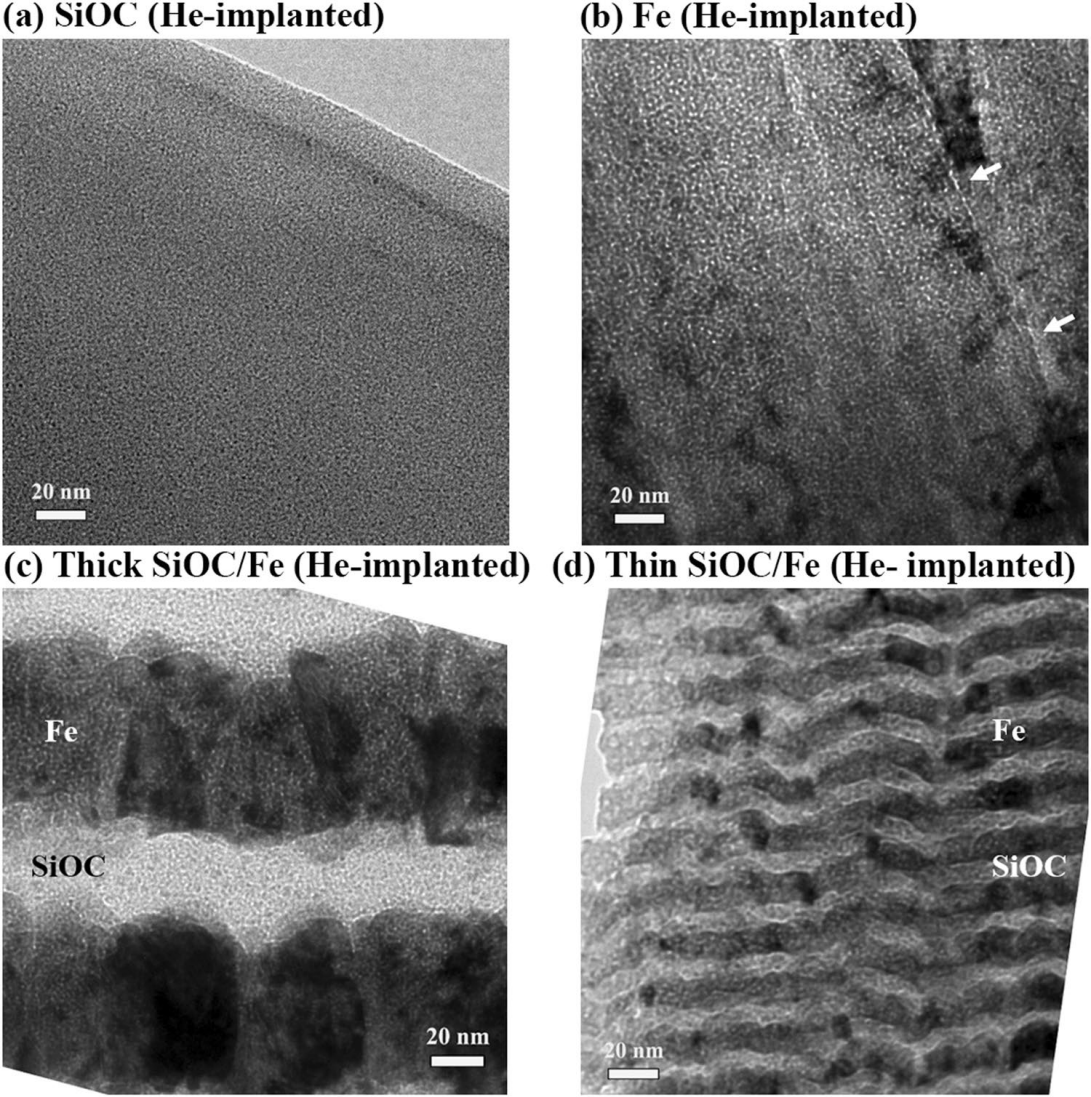
Under-focused high magnification cross-sectional bright-field TEM micrographs of the films after implantation. The micrographs were taken from regions where the He concentration was predicted by SRIM to be ~5 at. %.

To characterize the configuration of He bubbles present, high magnification TEM images of the implanted films were collected via through-focus imaging. Figure 4 shows the typical under-focused cross-sectional TEM micrographs of the films after implantation. The micrographs were taken from regions where the He concentration was predicted by SRIM to be ~5 at. %. The under-focused TEM micrographs were used to determine the average diameter of the He bubbles (*D*_*He*_) by taking into consideration the corresponding defocus (800 nm). Bubble density (*N*_*He*_, number of He bubbles per unit volume) was then determined by counting bubbles from the TEM micrographs and taking into consideration the thickness of the TEM specimen. Average spacing (center to center) between bubbles (*L*_*He*_) was estimated as ${L}_{He}=1/\sqrt{{N}_{He}{D}_{He}}$^2^. The obtained values of bubble diameter, density and average spacing are reported in Table 1.

The under-focused high magnification TEM micrograph of the SiOC film after implantation, presented in Fig. 4a, exhibits a maze-like pattern corresponding to the amorphous nature of the film. No He bubbles or voids are observed in the microstructure. This observation is consistent with our previous studies, where no He bubbles were observed in amorphous SiOC films subjected to an applied implantation dose of up to 113 at. %^13,19^. In contrast to the absence of He bubbles in the implanted SiOC film, the under-focused TEM micrograph of the implanted Fe film (Fig. 4b) shows He bubbles appearing as white dots surrounded by a dark Fresnel fringe. The bubbles are observed inside the grains and along the grain boundaries. Arrays of bubbles formed along grain boundaries are indicated by arrows in Fig. 4b. For the implanted Fe film, an average bubble diameter of 1 nm and density of 2.5 × 10^24^ m^−3^ were measured. The obtained bubble density is within the range of values typically reported for metals (10^18 ^– 10^25^ m^−3^)^6^. Figures 4c and 4d show under-focused high magnification TEM micrographs of the thick and thin SiOC/Fe nanolaminates after implantation, where He bubbles are observed in the Fe layers, but not in the SiOC layers. The amorphous/crystalline interfaces are also free from He bubbles. The average He bubble diameter was found to be similar for both nanolaminates (1 nm). The bubble density for both thick and thin SiOC/Fe nanolaminates (1.5 × 10^24^ m^−3^ and 0.9 × 10^24^ m^−3^, respectively) was found to be smaller than that for the Fe film. Also, the bubble density of the thin SiOC/Fe nanolaminate was less than the thick SiOC/Fe nanolaminate.

### Implantation-induced changes in mechanical response

Reduced elastic modulus and hardness of the films before and after implantation were evaluated by nanoindentation. To investigate the changes in reduced elastic modulus and hardness along the thickness of the films, the maximum indentation force (*P*_*max*_) was varied from 0.5 to 10 mN. A minimum of 10 indentations were performed at each load to obtain the average values and corresponding standard deviation of the reduced elastic modulus and hardness. During the experiments, the applied force and resulting penetration depth of an indenter were continuously recorded throughout a complete loading-unloading cycle. The recorded data was plotted as a force vs. penetration depth curve and the initial portion of the unloading curve was fit to a power law, which then allowed for the determination of the slope of the unloading curve at maximum penetration depth, i.e., stiffness (*S*). To obtain the elastic modulus and hardness of the films, it was necessary to find the projected area of contact between the indenter and the specimen (*A*_*p*_). SPM images of the cube corner impressions (presented in Supplementary Fig. S2) showed formation of pile-up around the edges of the impressions for all films except the implanted SiOC film. Formation of pile-up increases the depth over which the indenter and the specimen are in contact (contact depth, *h*_*c*_), and thus increases the contact area. The method of Oliver and Pharr^32^, typically used for determining the projected contact area, does not account for pile-up leading to the projected contact area calculated being smaller than the real area. This leads to an overestimation of the reduced elastic modulus and hardness. To account for pile-up in the analysis of the nanoindentation data, the projected contact area was measured directly from the post-indentation SPM images. The reduced elastic modulus (*E*_*r*_) and hardness (*H*) of the films were then determined using ${E}_{r}=\sqrt{\pi }\,S/2\sqrt{{A}_{P}}$ and H = *P*_*max*_/*A*_*p*_. Elastic modulus of the specimen (*E*) can be calculated from the reduced elastic modulus by $1/{E}_{r}=(1-{\nu }^{2})/E+(1-{\nu }_{i}^{2})/{E}_{i}$, where *v* is the Poisson’s ratio of the film and *v*_*i*_ and *E*_*i*_ are the Poisson’s ratio and elastic modulus of the indenter, in this case diamond (*v*_*i*_ = 0.07 and *E*_*i*_ = 1141  GPa)^32,33^. Since the Poisson’s ratio of the SiOC/Fe nanolaminates is unknown, results are reported in terms of reduced elastic modulus.

For indentations performed with a cube corner indenter, the contact depth varied between 20 – 760 nm. For contact depths greater than 100 nm (~1/10 of the film thickness), influence from the substrate on the reduced elastic modulus and hardness was unavoidable. Various treatments are available to account for the effect of the substrate on the elastic modulus and hardness of thin films. However, these treatments typically require a knowledge of the Poisson’s ratio of the material and are applicable to single layer films with uniform mechanical properties along their thickness^34,35^. Therefore, to avoid introducing uncertainties into calculations, the reduced elastic modulus and hardness data reported in this study were not subjected to any treatments. Although the reduced elastic modulus and hardness reported in this study are affected by the substrate, differences between the mechanical response of the as-deposited and implanted films can be attributed to the effects of implantation^36^. Nevertheless, some uncertainties may exist when comparing different films with one another^3^.

**Figure 5:**
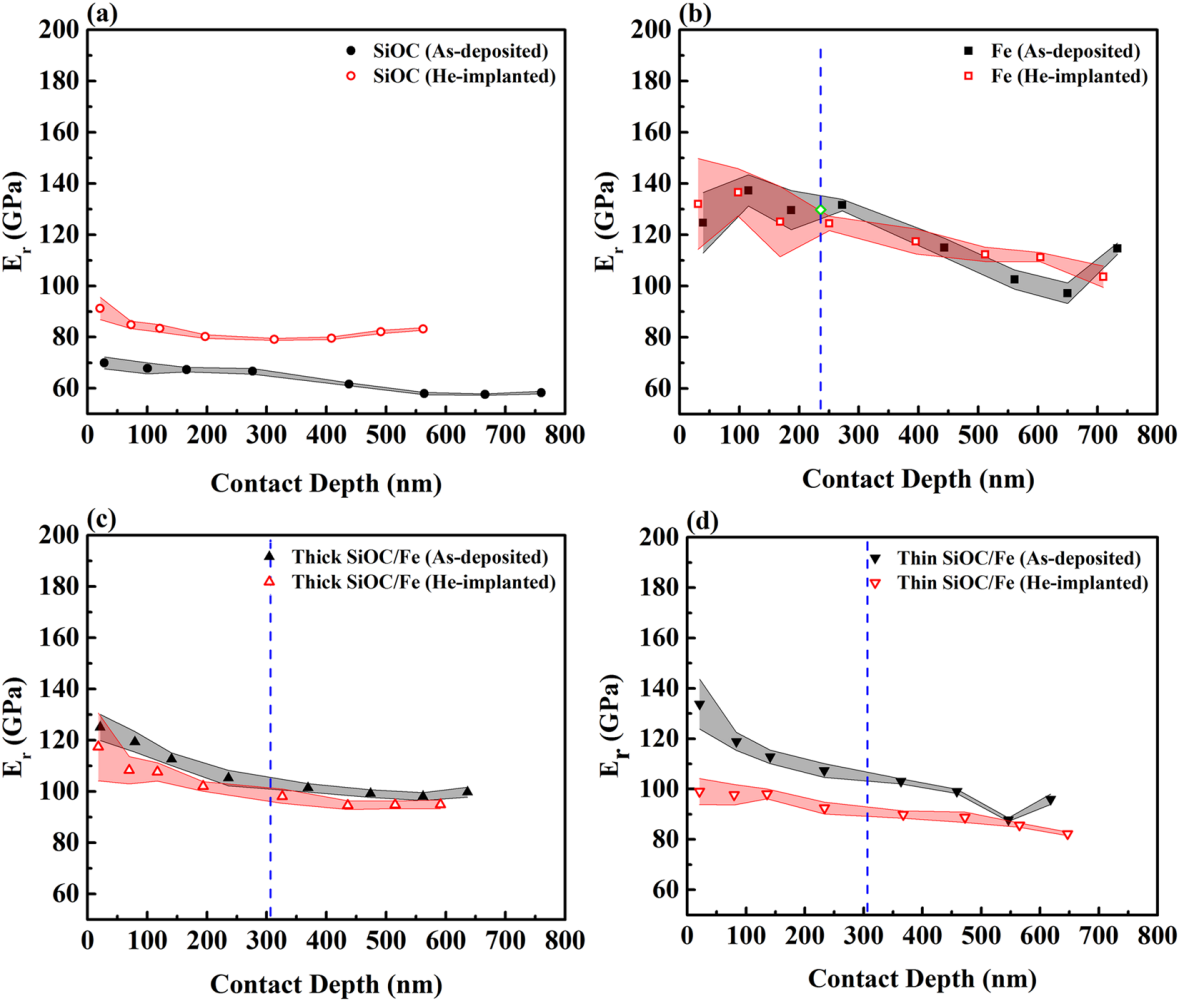
Average values of reduced elastic modulus of the films before and after implantation plotted as a function of contact depth. The dashed lines correspond to depths where the concentration of implanted He ions is highest. The open diamond shown in (**b**) corresponds to the reduced elastic modulus of the films after irradiation with Fe ions to a damage level of 2.5 dpa, reported in^23^.

**Figure 6:**
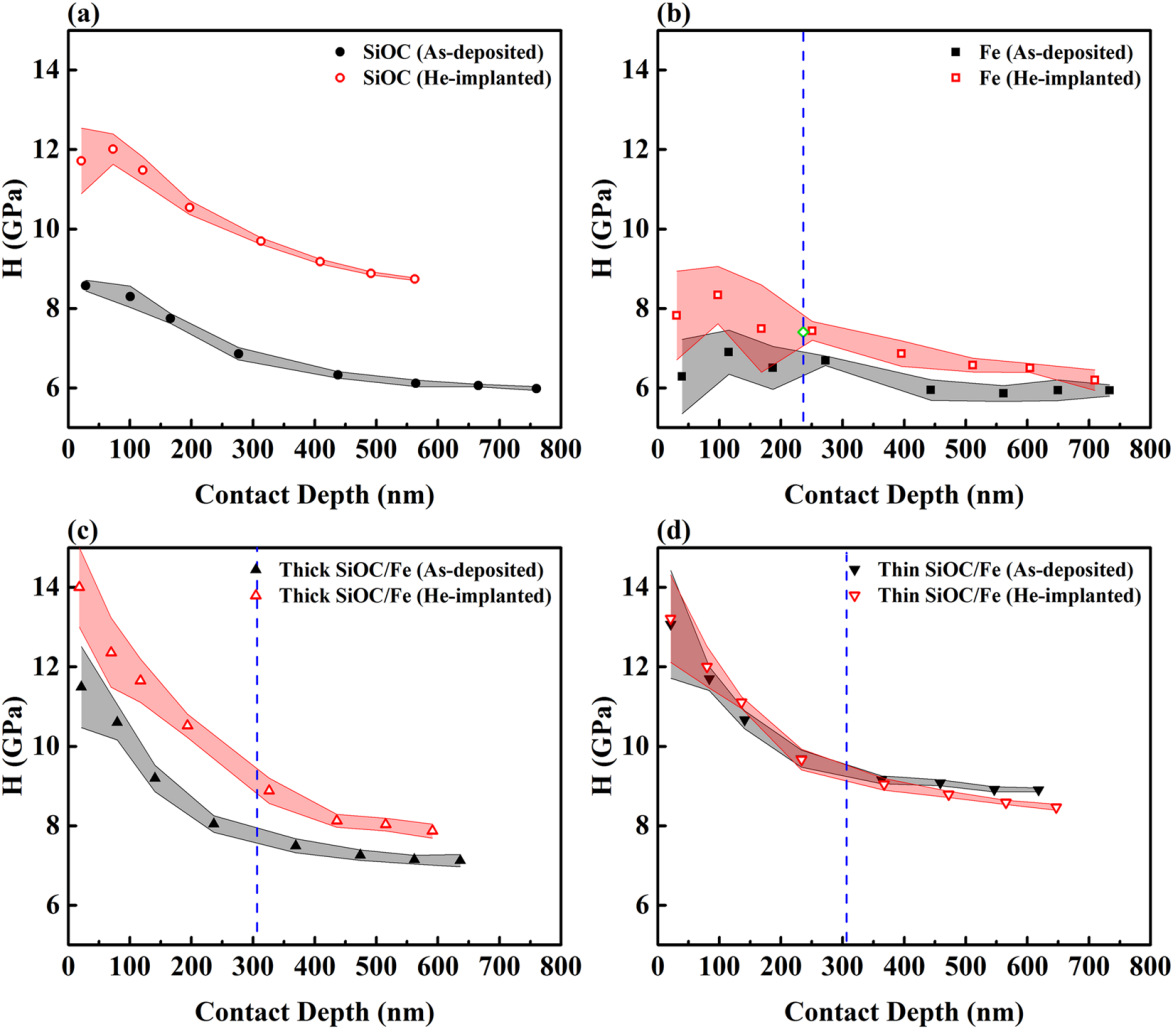
Average values of hardness of the films before and after implantation plotted as a function of contact depth. The dashed lines correspond to depths where the concentration of implanted He ions is highest. The open diamond shown in (**b**) corresponds to the hardness of the films after irradiation with Fe ions to a damage level of 2.5 dpa, reported in^23^.

Figures 5 and 6 show average values of reduced elastic modulus and hardness of the films before and after implantation plotted as a function of contact depth. Except for the SiOC film, which based on the TEM results did not contain any bubbles, the depths corresponding to the maximum concentration of implanted He ions are marked on the plots. For almost all the films, the reduced elastic modulus and hardness decreased with increasing contact depth, which shows the influence of the substrate. The indentation size effect is also seen in the hardness results. Additionally, the higher Ra values of the Fe films compared to the SiOC film and the thick and thin nanolaminates results in more variation of the reduced elastic modulus and hardness of the Fe films below a contact depth of 100 nm^33^. Discrete displacement bursts, sudden increases in penetration depth at a relatively constant force during loading, are most often attributed to the onset of inhomogeneous plasticity through nucleation of dislocations or shear bands or breakthrough of a thin film^37^. The force vs. penetration depth curves obtained from as-deposited and implanted films were continuous and free from displacement bursts, indicating homogenous deformation in the films without the formation of cracks. This was further supported by the absence of cracks in the SPM images of the cube corner impressions (Supplementary Fig. S2).

## Discussion

Our microstructural studies demonstrated that SiOC retained its amorphous structure after implantation. Furthermore, no He bubbles were observed in the under-focused TEM micrographs of the implanted SiOC film. Amorphous SiOC consists of structural tetrahedral units of SiO_4-x_C_x_ (x = 0 – 4). According to the random bonding model (RBM), Si atoms randomly bond with O and C atoms to form a homogenous network. Because of the relatively low deposition temperature in magnetron sputtering, as-deposited atoms lack sufficient kinetic energy to reach a thermodynamic equilibrium state and are in a metastable state^38^. Formation of collision cascades and thermal spikes during irradiation give rise to increased atomic mobility and allow the material to undergo structural relaxation, which transforms the as-deposited metastable amorphous structure into a more relaxed state. This relaxation process is accompanied by a reduction in free volume and structural rearrangements^13,38^. Our previous studies demonstrated that although He bubbles were not retained in amorphous SiOC, implantation led to a reduction in the number of Si‒O bonds and an increase in the number of Si‒C and C‒O bonds^13^. The absence of He bubbles in the microstructure is a possible indication of an interstitial-like diffusion mechanism at work, where He ions diffuse through the free volume present in the amorphous structure^13^.

For the Fe film and the nanolaminates, irradiation damage led to Fe grain growth. According to Table 1, the average size of the Fe grains was similar in the as-deposited Fe film and the nanolaminates. After implantation however, the average size of the grains in the Fe film was almost three times larger than the thick SiOC/Fe nanolaminate. For the thin SiOC/Fe nanolaminate, changes in the average size of the Fe grains was minor. Generation of point defect and thermal spikes during ion irradiation, which leads to increased atomic mobility, is the underlying mechanism responsible for grain growth. A plausible explanation for the lower magnitude of grain growth in the nanolaminates compared to the Fe film is the annihilation of point defects at amorphous/crystalline interfaces and a subsequently lower concentration of mobile atoms^21^.

Another consequence of implantation in the Fe film and the nanolaminates was the formation of He bubbles, as evidenced by the TEM micrographs. Extensive TEM studies revealed that the depth profile of He bubble density follows a trend similar to that of the implanted He ion concentration obtained by SRIM (shown in Figs. 1b and 1c). Additionally, the depth corresponding to the maximum bubble density was found to coincide with that of maximum He ion concentration (236 nm for the Fe film and 306 nm for the thick and thin SiOC/Fe nanolaminates). Average bubble diameter, maximum bubble density, and average spacing between bubbles are reported in Table 1. The average bubble diameter was the same in the Fe film and the nanolaminates, which shows that bubble size is independent of the thickness of the Fe layers. However, the maximum density of He bubbles in the Fe film was 2 – 3 times higher than the nanolaminates. Since He bubbles are formed from He-vacancy clusters, a lower density of He bubbles suggests an overall reduction in the concentration of vacancies present in the system. It is generally accepted that irradiation-induced defects tend to migrate to interfacial regions (e.g., grain boundaries and interfaces) that effectively act as sinks^1,2,4,5,7^. Therefore, it appears that amorphous/crystalline interfaces facilitate the annihilation of defects created during implantation in the nanolaminates. This is further supported by the bubble density of the thin SiOC/Fe nanolaminate being less than the thick SiOC/Fe nanolaminate. By reducing the thickness of individual layers, the total area of amorphous/crystalline interfaces per unit volume increases, which assists defect annihilation. Similar observations have also been reported in crystalline/crystalline nanolaminates^1,2,4,5,9,10^.

The XRD results of the thick SiOC/Fe nanolaminate after implantation indicated a shift to smaller 2θ values for the bcc Fe peaks corresponding to out-of-plane lattice expansion. Formation of pressurized He bubbles is a plausible explanation for the lattice expansion based on the point source dilatation mechanism^2,5^. Since the film is constrained by the substrate, lattice expansion in the normal direction induces a biaxial compressive elastic stress in the plane of the film^1,2^, which could affect the mechanical properties. He bubble-induced lattice expansion has been observed in other systems, where the magnitude of expansion was seen to be proportional to bubble density^1,2,4,5^. Since bubble density changes along the thickness of the films, a quantitative evaluation of lattice expansion was not attempted from the XRD results. However, a qualitative comparison between the XRD patterns of the two SiOC/Fe nanolaminates (Figs. 2c and 2d) demonstrated that, unlike the thick nanolaminate, no shift was observed in the XRD patterns of the thin nanolaminate. This indicates that decreasing the thickness of the individual layers results in a reduction or disappearance of lattice expansion^2,5^ and is consistent with the maximum He bubble density of the thin nanolaminate (0.9 × 10^24^ m^−3^) being lower than the thick nanolaminate (1.5 × 10^24^ m^−3^).

The average values of reduced elastic modulus and hardness over a range of contact depths measured from films before and after implantation are shown in Figs. 5 and 6. Prior to implantation, at a contact depth of ~100 nm, reduced elastic moduli of 67.8 and 137.3 GPa were obtained for the SiOC and Fe films, respectively. The reduced elastic moduli of the as-deposited thick and thin SiOC/Fe nanolaminates at the same contact depth (119.3 and 118.9 GPa, respectively) were between that of the SiOC and Fe films. On the other hand, the hardness of the as-deposited thick and thin SiOC/Fe nanolaminates at a contact depth of ~100 nm (10.6 and 11.7 GPa, respectively) was higher than the hardness of the SiOC and Fe films (8.3 and 6.9 GPa, respectively). These observations are consistent with our previous study where a Berkovich indenter was used for indentations and the contact depth was limited to 1/10 of the film thickness^23^. The enhanced hardness of the nanolaminates has been attributed to plastic co-deformation of the SiOC and Fe layers that delays the failure of the amorphous layers^23^. Also, a comparison between the hardness of the as-deposited thick and thin SiOC/Fe nanolaminates suggests the hardness of the nanolaminates increases with decreasing layer thickness. Since the thickness of individual Fe layers in thick and thin SiOC/Fe nanolaminates is in the range of 10 – 100 nm, confined layer slip (CLS) is the dominant deformation mechanism^39^. The shear stress required to propagate a dislocation loop confined to a layer is inversely proportional to layer thickness^11^. Therefore, the shear stress, and thus the strength and hardness, of the nanolaminate films increase with decreasing the layer thickness.

As shown in Fig. 5a, implantation resulted in an increase in the reduced elastic modulus of the SiOC film over all contact depths investigated. Implantation-induced microstructural evolution is a plausible explanation for the increase in reduced elastic modulus of the SiOC film. This is further discussed below. As seen in Fig. 5b, He implantation had minimal effect on the reduced elastic modulus of the Fe film at contact depths ≤450 nm. A comparison between the change in reduced elastic modulus of the He-implanted Fe film with that reported in our previous study for an Fe film subjected to irradiation with 3.5 MeV Fe ions^23^ also showed a similar effect. Linear interpolation of the data of reduced elastic modulus obtained as a function of dpa from^23^, results in a reduced elastic modulus of 129.8 GPa for the Fe film irradiated with Fe ions to a damage level of 2.5 dpa at a contact depth of ~100 nm. This value of reduced elastic modulus (shown as an open diamond in Fig. 5b) is comparable to the reduced elastic modulus of the He-implanted Fe film at a depth of ~236 nm, which corresponds to both a damage level of 2.5 dpa and 5 at. % He implantation. Hence, the reduced elastic modulus of the Fe film in the present study does not depend on the presence of He bubbles. Similar observations have been reported for a W–Ta alloy, where no significant change in elastic modulus was observed after implantation with W ions^40^. As shown in Figs. 5c and 5d, He implantation resulted in a reduction in reduced elastic moduli of both the thick and thin nanolaminates. Further work is required to determine the underlying reasons for the decrease in the reduced elastic modulus of the nanolaminates. A comparison between Figs. 5c and 5d also demonstrates that implantation-induced reduction in reduced elastic modulus of the thick nanolaminate (3 – 9%) is smaller than the thin nanolaminate (13 – 26%, except at a contact depth of ~550 nm). The presence of a compressive elastic stress in the plane of the thick nanolaminate (induced by the out-of-plane lattice expansion, evidenced by the XRD results) could be a plausible explanation for the smaller drop in reduced elastic modulus of this film^41^. The underlying reason for the anomaly observed at a contact depth of ~550 nm in the thin SiOC/Fe nanolaminate is unclear.

In addition to the increase in reduced elastic modulus, irradiation of the SiOC film with He ions led to an increase in hardness for all contact depths (Fig. 6a). The absence of He bubbles, evidenced by the TEM results, rules out any influence from bubbles on the mechanical response. Increased reduced elastic modulus and hardness of the implanted SiOC film can likely be explained by microstructural evolutions, where implantation leads to a reduction in the number of Si‒O bonds and an increase in the number of Si‒C and C‒O bonds^13^. Due to these structural rearrangements, the possibility of finding C-rich SiO_4-x_C_x_ tetrahedral units in the implanted SiOC film is higher than in the as-deposited film. Formation of C-rich SiO_4-x_C_x_ tetrahedral units leads to an increased bond density and thus creates a stiffer and more constrained atomic structure with a higher elastic modulus and hardness^38,42,43^. Also, an increase in the number of C-rich SiO_4-x_C_x_ tetrahedral units increases the possibility of finding tetrahedral units that are connected via Si‒C‒Si bridging bonds rather than Si‒O‒Si bonds. Since the C atoms form four bonds with the Si atoms, rather than two bonds for O, the Si‒C‒Si bridging bonds are much more rigid than the Si‒O‒Si bonds^38^. This further increases the elastic modulus and hardness of the SiOC film after implantation.

As seen in Fig. 6b, He implantation of the Fe film led to an increase in hardness for all the contact depths. Implantation/irradiation-induced hardening of crystalline metals is a result of the interactions between glide dislocations and generated defects, such as He bubbles and interstitial loops^1,3–5^. Previous studies on stainless steel have shown at approximately 1 at. % He concentration, dislocations can be pinned by He bubbles in the lattice^3^. Therefore, He bubble strengthening is a possible mechanism for the increased hardness of the Fe film. Molecular dynamic simulations of Fe implanted with He has demonstrated that the barrier strength of He bubbles depends on the He/vacancy ratio (*R*), where bubbles with a ratio of 1 – 2 are weak obstacles for dislocation motion^5,44^. To determine the barrier strength of the He bubbles, the equilibrium pressure of He bubbles (*P*) was calculated by *P* = 4*γ*/*D*_*He*_ where *γ* is the surface energy of the bubble. Bubble pressure (in GPa) was also estimated by the exponential approximation of the equation of state (EOS) for He expressed as $P=4.83\times {10}^{7}\exp (5.15\times {10}^{-23}\frac{{N}_{0}}{V})$, where *N*_0_ is Avogadro’s number and *V* is the He molar volume^45^. The obtained He molar volume, in turn, was used to find the He/vacancy ratio in each bubble based on $V=\frac{{a}_{Fe}^{3}\times {N}_{0}}{2\times R}$, where *a*_*Fe*_ is the lattice parameter of Fe (0.286 nm)^23^. Using a surface energy of 2.0 J/m^2^ ^7,46^, an equilibrium bubble pressure of 6.7 GPa was obtained for the Fe films, which corresponds to a He molar volume of 6.3 cm^3^/mol and a He/vacancy ratio of 1.1. These values are comparable to those reported for nanocrystalline Fe subjected to He implantation^7^. Therefore, the bubbles formed in the Fe film can be considered as weak obstacles for dislocation motion. For weak obstacles, the irradiation-induced increase in yield strength (Δ*σ*) is described by the Friedel-Kroupa-Hirsch (FKH) model expressed as ${\rm{\Delta }}\sigma =\frac{1}{8}MGb{D}_{He}{N}_{He}^{2/3}$, where M is the Taylor factor (3.05 for bcc metals), *G* is the shear modulus (calculated as 54 GPa from nanoindentation results at a contact depth of 100 nm assuming a Poisson’s ratio of 0.29), and b is the Burgers vector (0.248 nm)^1,3,5,7,44^. Using the diameter and maximum density of the He bubbles, the implantation-induced increase in yield strength based on the FKH model was calculated to be 0.1 GPa, which corresponds to a 0.3 GPa increase in hardness using a Tabor factor of 2.7^7^. Changes in the hardness, at a depth that corresponds to 5 at. % He concentration, were estimated from the hardness profiles shown in Fig. 6b and are reported in Table 1. The increase in hardness calculated from the FKH model accounts for ~40% of the increase in hardness measured by nanoindentation. To validate the prediction of the FKH model, hardness of the He-implanted Fe film was compared with that reported in our previous study for an Fe film subjected to irradiation with 3.5 MeV Fe ions^23^. Linear interpolation of the data of hardness as a function of dpa from^23^, results in a hardness of 7.4 GPa for the Fe film irradiated with Fe ions to a damage level of 2.5 dpa at a contact depth of ~100 nm. This hardness value (shown as an open diamond in Fig. 6b) is comparable to the hardness of the He-implanted Fe film at a depth of ~236 nm, which corresponds to both a damage level of 2.5 dpa and maximum concentration of implanted He ions. Hence, it is concluded that within the uncertainty of our measurements there is no measurable effect from the interaction of dislocations with He bubbles on the increase in hardness. Although the hardness of the Fe-irradiated and He-implanted Fe films was recorded at different contact depths (~100 nm and 236 nm, respectively), the influence of the substrate and the indentation size effect are not expected to significantly affect this comparison because, as seen in Fig. 6b, hardness of the as-deposited and He-implanted Fe films does not change significantly with contact depth. Interaction of dislocations with interstitial loops is also a plausible mechanism for hardening. Interstitial loops are typically treated as strong barriers to the glide of dislocations. For strong obstacles, a dispersed barrier model is used to describe the increase in yield strength ${\rm{\Delta }}\sigma =M\alpha Gb\sqrt{{D}_{d}{N}_{d}}$, where *M*, *G*, and *b* carry the same physical meanings as defined in the FKH model, *D*_*d*_ and *N*_*d*_ are the diameter and density of interstitial loops, and *α* is the barrier strength (typically 0.45). Direct experimental determination of loop density via microscopy is challenging. The loop density can be estimated indirectly from the experimentally measured increase in hardness (0.8 GPa). Assuming a loop diameter of 5 nm^1^, our analysis implied an interstitial loop density of 4.2 × 10^22^ m^−3^, which is comparable with values reported for neutron or proton irradiated Fe^47–49^.

As seen in Fig. 6c, He implantation of the thick SiOC/Fe nanolaminate led to an increase in hardness. Based on our previous study^23^, the SiOC and Fe layers with individual layer thicknesses of 72 ± 10 nm deform collectively and homogenously to accommodate the strain applied to the SiOC/Fe nanolaminate. During deformation, single dislocation loops form inside the Fe layers and propagate parallel to the interfaces (CLS mechanism). Since localized shear flow is inhibited by geometrical constraints, the SiOC layers can also undergo homogenous deformation through plastic flow and/or compaction^23^. According to Table 1, the average spacing of He bubbles in the thick SiOC/Fe nanolaminate (25 nm) was smaller than the thickness of individual Fe layers (80 nm). Therefore, when a dislocation loop becomes mobile inside the Fe layers, it may interact with He bubbles before reaching the interface^2,5^ and thus lead to hardening. For the thick nanolaminate, He bubble pressure, molar volume, and He/vacancy ratio were found to be 7.3 GPa, 6.2 cm^3^/mol, and 1.1, respectively. Therefore, the He bubbles can be considered as weak barriers for dislocation motion. Using the FKH model, implantation-induced hardening caused by He bubbles was estimated to be ~0.2 GPa, which accounts only for ~15% of the experimental value. Unlike the case of the Fe films, we are unable to validate the prediction of the FKH model through a comparison between the hardness of the He-implanted thick SiOC/Fe nanolaminate with that reported in our previous study for a SiOC/Fe film with individual layer thicknesses of 72 ± 10 nm subjected to irradiation with 3.5 MeV Fe ions^23^. This is because the reduced elastic moduli of the Fe-irradiated and He-implanted Fe films were measured at different contact depths (~100 nm and 306 nm, respectively) and, as seen in Fig. 6c, the hardness of the thick SiOC/Fe nanolaminate decreases significantly with increasing the contact depth. In addition to He bubbles, other plausible factors contributing to hardening in this nanolaminate are irradiation-induced formation of interstitial loops and hardening of the individual SiOC layers. Our nanoindentation results showed an increase in the hardness of the SiOC film after implantation with He. The effects of reducing the thickness of SiOC (from 1 μm in the single layer film to 60 and 14 nm in the thick and thin nanolaminates, respectively) on its deformation mechanism have not been reported. However, implantation-induced hardening of the SiOC layers in the thick nanolaminate is not implausible, which could further increase the hardness. Further investigation in this area is warranted.

Similar to the Fe and thick SiOC/Fe films, the He/vacancy ratio for the thin nanolaminate was also found to be 1.1. Therefore, He bubbles formed in the thin nanolaminate can also be considered as weak obstacles for dislocation motion. However, based on Fig. 6d, the hardness of the implanted thin SiOC/Fe nanolaminate is comparable to the as-deposited film. A comparison between the changes in hardness estimated from the nanoindentation experiments and those calculated by the FKH model (see Table 1) showed that although the nanoindentation results demonstrated a slight decrease in hardness, the FKH model predicted an increase. In other words, it appears that the FKH model fails to accurately describe the mechanical response of the thin SiOC/Fe nanolaminate. This discrepancy can be explained by the average bubble spacing (32 nm) being almost two times larger than the thickness of individual Fe layers in this nanolaminate (14 nm). This makes it unlikely for dislocation loops to interact with He bubbles before reaching the interface. As a result, although He bubbles are formed in the thin SiOC/Fe nanolaminate, they do not lead to hardening. The plastic deformation of the thin SiOC/Fe nanolaminate before and after implantation is dominated by the stress necessary to transmit dislocations across the interface^2,5^.

## Conclusions

The evolution of microstructure and mechanical properties of 1 μm thick amorphous SiOC, nanocrystalline Fe, and SiOC/Fe nanolaminate films subjected to 50 keV He^+^ implantation at room temperature was systematically investigated. He bubbles were observed in all the films except SiOC. A lower bubble density was obtained for the SiOC/Fe nanolaminates compared to the Fe film. The average size of Fe grains after implantation was also smaller in the SiOC/Fe nanolaminates compared to the Fe film. These observations suggest that amorphous/crystalline interfaces act as efficient defects sinks, promoting interstitial and vacancy recombination and mitigating He bubble accumulation and grain growth. The SiOC/Fe nanolaminates also remained structurally stable after implantation as the amorphous/crystalline interfaces remained intact.

He implantation was seen to result in an increased hardness in the SiOC and Fe films. The hardening of the SiOC film was attributed to microstructural evolution and an increase in the number of Si‒C and C‒O bonds. The FKH model predicted an increase in the hardness of the Fe film due to the formation of He bubbles. However, a comparison between the hardness of He-implanted Fe film with that reported in our previous study for an Fe film subjected to irradiation with 3.5 MeV Fe ions, demonstrated that within the uncertainty of our measurements there is no measurable effect from the interaction of dislocations with He bubbles on the increase in hardness. Formation of interstitial loops as a result of irradiation damage is a plausible mechanism that could contribute to hardening in the Fe film. For the SiOC/Fe nanolaminates, hardening was only observed in the nanolaminate with alternating layers of 60 nm thick SiOC and 80 nm thick Fe, in which the average spacing between the He bubbles was comparable or smaller than the thickness of individual layers. The increased hardness was found to be only partially resulted from the interactions of dislocations with He bubbles. Other plausible factors contributing to hardening in this nanolaminate are hardening of the individual SiOC layers and irradiation-induced formation of interstitial loops. When the thickness of the individual SiOC and Fe layers was reduced to 14 nm, hardness of the SiOC/Fe nanolaminate remained unchanged after implantation. The SiOC/Fe nanolaminate with individual layer thicknesses of 14 nm is a promising candidate for applications that require irradiation tolerant materials since its microstructure was stable and its hardness remained essentially unchanged after implantation.

## Methods

### Specimen preparation

A single layer SiOC film, a single layer Fe film, a SiOC/Fe nanolaminate film with alternating layers of 60 nm thick SiOC and 80 nm thick Fe (referred to as thick SiOC/Fe), and a SiOC/Fe nanolaminate film with alternating layers of 14 nm thick SiOC and 14 nm thick Fe (referred to as thin SiOC/Fe) were used in this study. Radio frequency (RF) magnetron sputtering was used to synthesize amorphous SiOC from SiO_2_ and SiC targets with purities of 99.995% and 99.5%, respectively. The nominal composition of the as-deposited SiOC was 30 at. % Si, 40 at. % O, and 30 at. % C^19,50^. Direct current (DC) magnetron sputtering was used to synthesize α-Fe from an Fe target with a purity of 99.95%. All films were deposited on oxidized Si wafer substrates and had nominal thicknesses of 1 μm. For the SiOC/Fe nanolaminates, the last layer deposited on the surface was SiOC to prevent possible oxidation of the Fe and also to obtain a lower surface roughness.

### Ion implantation

The as-deposited films were subjected to 50 keV He^+^ implantation at room temperature. To obtain a 5 at. % peak concentration of implanted He ions, fluences of 6.8 × 10^16^, 6.5 × 10^16^, and 7.0 × 10^16^ ion/cm^2^ were used for the SiOC, Fe, and SiOC/Fe films, respectively. Stopping and Range of Ions in Matter (SRIM)-2008^25^ was used to calculate the simulated depth profiles of the implanted ion concentration and irradiation damage. For the simulations, the SiOC/Fe nanolaminates were considered as a uniform layer of amorphous material with a nominal composition of Fe_13.3_Si_3_O_4_C_3_ and a density of 5.3 g/cm^3^.

### Atomic force microscopy

A commercial Bruker atomic force microscope operating in tapping mode was used to examine the surface topography and measure the average surface roughness (Ra) of the films before and after implantation.

### Characterization of structural properties

A Bruker-D8 Discover X-ray diffractometer was used to characterize the crystallographic structure of the films before and after implantation. A monochromatic Cu Kα_1_ radiation, with a wavelength of 0.1540562 nm, was used for the experiment. Except for the SiOC film, the XRD patterns were collected using the conventional θ/2θ scanning configuration. For the SiOC films, the incident angle of the X-ray was held constant at the lowest value possible for the instrument (7.5°) to minimize the substrate peaks and enhance any signal from SiOC. An FEI Tecnai G2 F20 transmission electron microscope (TEM) was used to examine the cross-sectional microstructure of the films before and after implantation. The cross-sectional specimens used for TEM were prepared through grinding, polishing, and ion milling.

### Characterization of mechanical properties

A force-controlled Hysitron Triboindenter was used to study the mechanical response of the as-deposited and implanted films. Indentations were performed using a diamond cube corner indenter. After reaching thermal equilibrium in the instrument enclosure, the films were indented over a range of maximum forces (0.5, 1, 2, 4, 6, 8, or 10 mN) using a loading rate of 0.2 mN/s. The force was then held constant at the maximum for 60 s to allow any time dependent plastic effects to diminish. During unloading, the force was reduced to 10% of the maximum force in 10 s, held constant for 60 s to measure thermal drift, and finally reduced to zero in 2 s. Immediately after the final unloading, the diamond indenter was used as a scanning probe tip to study the cube corner impressions.

## Supplementary information

Zare et al Supplementary Information

## Data Availability

All data is available from the authors upon reasonable request.
