# Supplementary material for: Mechanical Response of He-Implanted Amorphous SiOC/Crystalline Fe Nanolaminates
Source: Sci Rep. 2019 Mar 18;9:4759. doi: 10.1038/s41598-019-41226-w (PMC6423206; doi:10.1038/s41598-019-41226-w)
Supplement: Supplementary file 1 — Zare et al Supplementary Information [file 41598_2019_41226_MOESM1_ESM.pdf]

Mechanical Response of He-Implanted Amorphous SiOC/Crystalline Fe Nanolaminates

A. Zare[1], Q. Su[2], J. Gigax[3], T.A. Harriman[1], M. Nastasi[2,4,5], L. Shao[3], D.A. Lucca[1 *]

[1]School of Mechanical and Aerospace Engineering, Oklahoma State University, Stillwater, OK 74078, USA.
[2]Nebraska Center for Energy Sciences Research, University of Nebraska-Lincoln, Lincoln, NE 68583, USA.
[3]Department of Nuclear Engineering, Texas A&M University, College Station, TX 77840, USA.
[4]Department of Mechanical and Materials Engineering, University of Nebraska-Lincoln, Lincoln, NE 68583, USA.
[5]Nebraska Center for Materials and Nanoscience, University of Nebraska-Lincoln, Lincoln, NE 68588, USA.
Correspondence and requests for materials should be addressed to D.A.L. (email: lucca@okstate.edu)

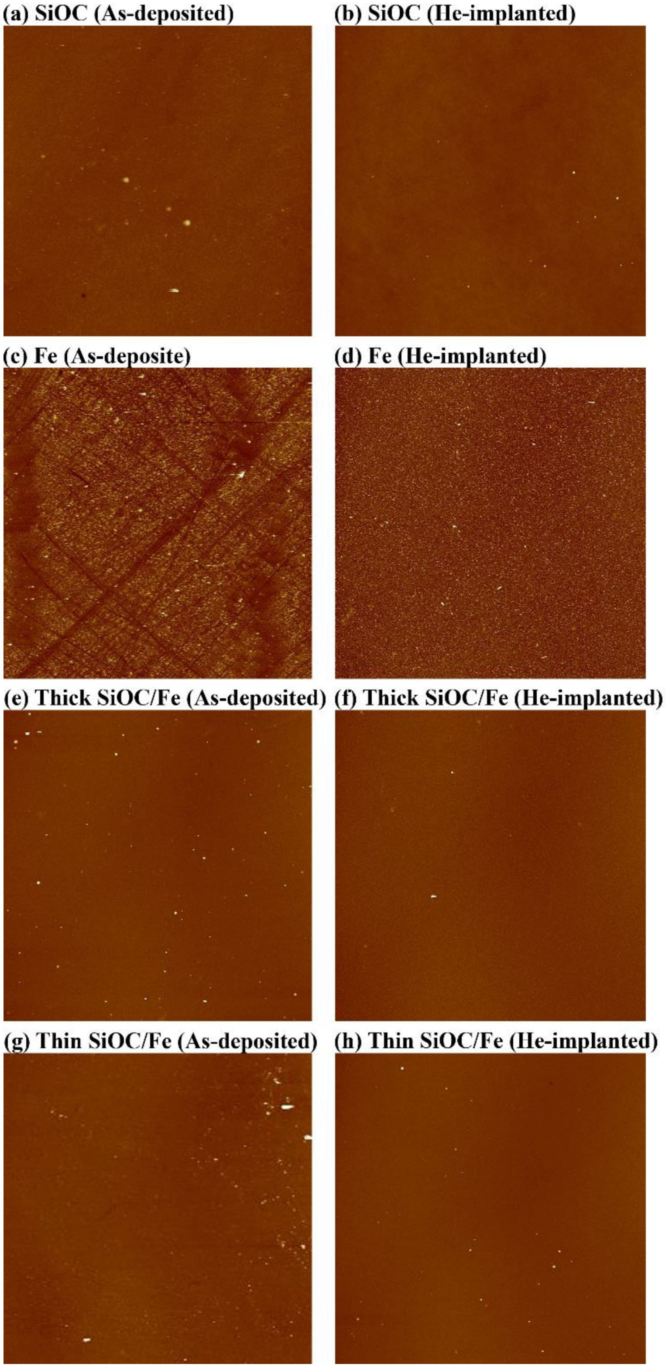

Figure S1. Surface topography images of the as-deposited and implanted films, obtained by AFM. All the scan areas are 80×80 μm^2 and the height scales are 200 nm.

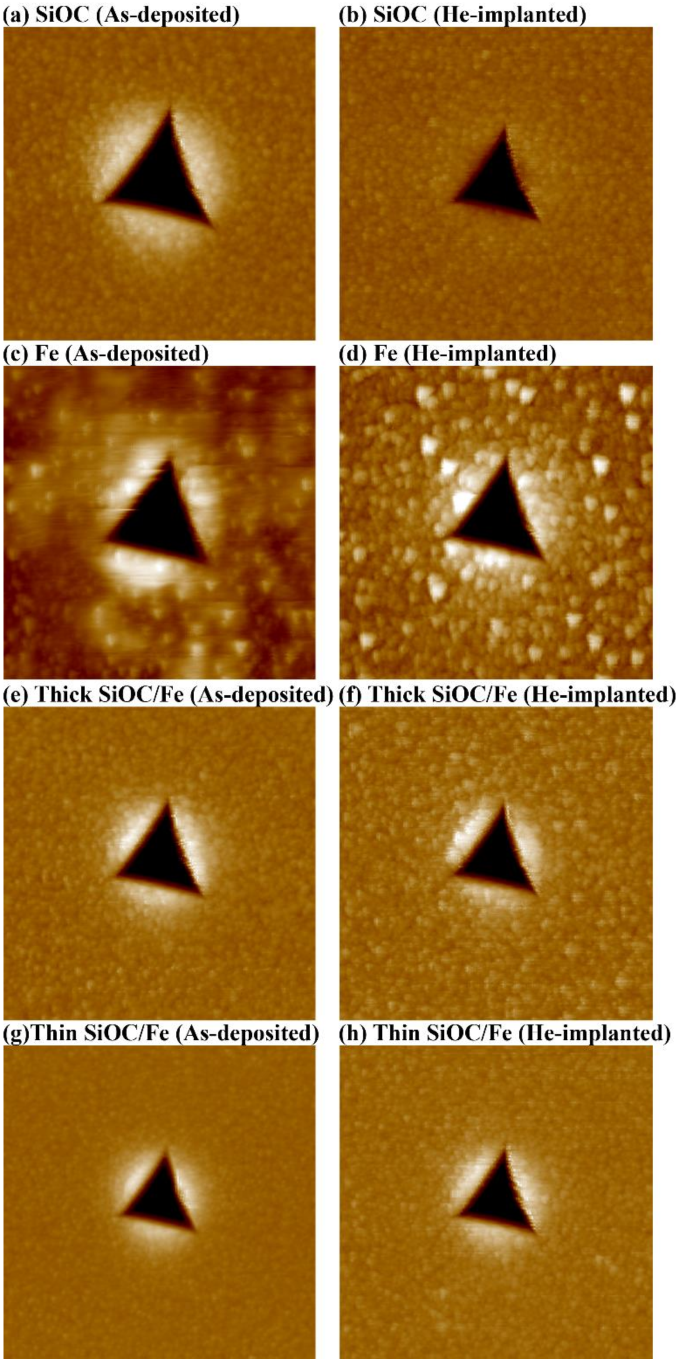

Figure S2. Post-indentation SPM images of residual impressions from indentations performed with the cube corner indenter at a maximum force of 10 mN. All the scan areas are $5 \times 5\ \mu m^2$ and the height scales are 200 nm.
